# Supplementary material for: Map of synthetic rescue interactions for the Fanconi anemia DNA repair pathway identifies USP48
Source: Nat Commun. 2018 Jun 11;9:2280. doi: 10.1038/s41467-018-04649-z (PMC5996029; doi:10.1038/s41467-018-04649-z)
Supplement: Supplementary file 1 — Supplementary Information [file 41467_2018_4649_MOESM1_ESM.pdf]

Map of synthetic rescue interactions for the Fanconi anemia DNA repair pathway identifies USP48

Velimezi G., Robinson-Garcia L., *et al.*

Antibody	Species	Company	Catalog number	Technique	Dilution
FANCC-8F3	Mouse	Merck-Millipore	MABC524	WB	1:250
FANCG	Rabbit	Novus	NB100-2566	WB	1:2000
FANCA	Rabbit	Bethyl	301-980A-T	WB	1:1000
FANCD2	Rabbit	Abcam	ab108928	WB	1:2000
FANCD2	Mouse	Santa Cruz Biotech	sc-20022	IF	1:100
FANCI	Rabbit	Bethyl Labs	A301-254	WB	1:1000
USP48	Rabbit	Bethy Labs	A301-190	WB	1:4000
ß-Actin	Rabbit	Sigma	A5060	WB	1:5000
BRCA1 D-9	Mouse	Santa Cruz Biotech	sc-6954	IF	1:500
RPA32	Mouse	Abcam	ab2175	WB / FACS	1:5000 / 1:500
Rad51	Rabbit	Santa Cruz Biotech	sc-8349	IF	1:100
GFP	Rabbit	ThermoFisher Scientific	A-6455	IF	1:800
GFP	Mouse	Roche Applied Science	11814460001	WB	1:1000
HA-tag	Mouse	Covant Research Products	MMS-101R	WB	1:500
Flag M2	Mouse	Sigma-Aldrich	F1804	WB	1:2000
FK2	Mouse	Enzo Life Sciences Ltd	BML-PW8810	WB	1:1000
γH2AX S139 JBW301	Mouse	Merck-Millipore	05-636	IF	1:1000

Supplementary Table 1. Antibody list indicating Catalog number, technique and conditions used. WB: Western Blot assay, IF: immunofluorescence assay, FACS: Fluorescence-activated cell sorting assay.

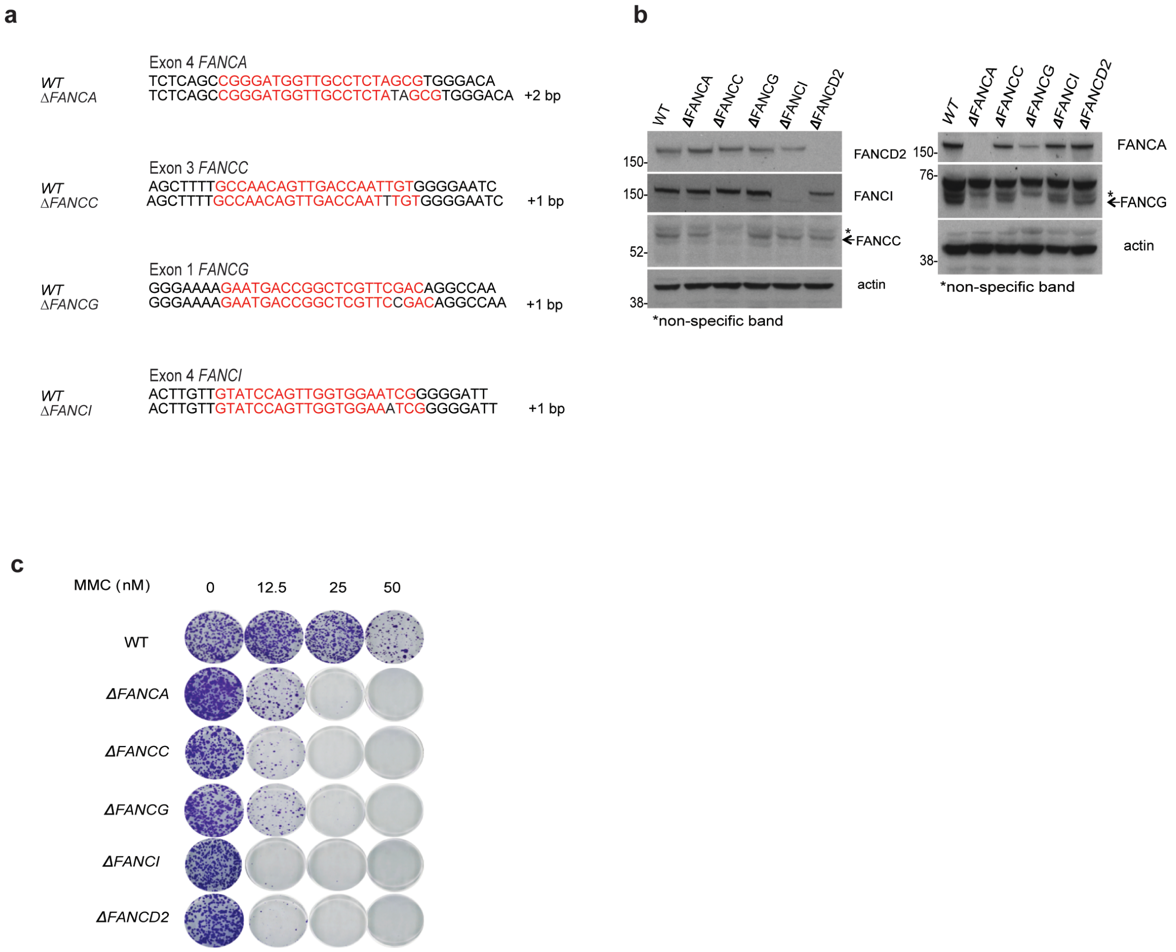

Supplementary Figure 1. CRISPR-Cas9 mediated generation of HAP1 FA-defective cells. (a) CRISPR-Cas9-mediated mutation of FA genes in HAP1 cells. Red sequences in wild-type (WT) correspond to the guide RNAs (gRNAs) used. (b) Immunoblot for FANCA, FANCC, FANCG, FANCI, FANCD2 and actin. Asterisk (*) denotes non-specific band. (c) Colony formation of the FA-defective HAP1 cell lines 7 days after treatment with MMC at the indicated doses.

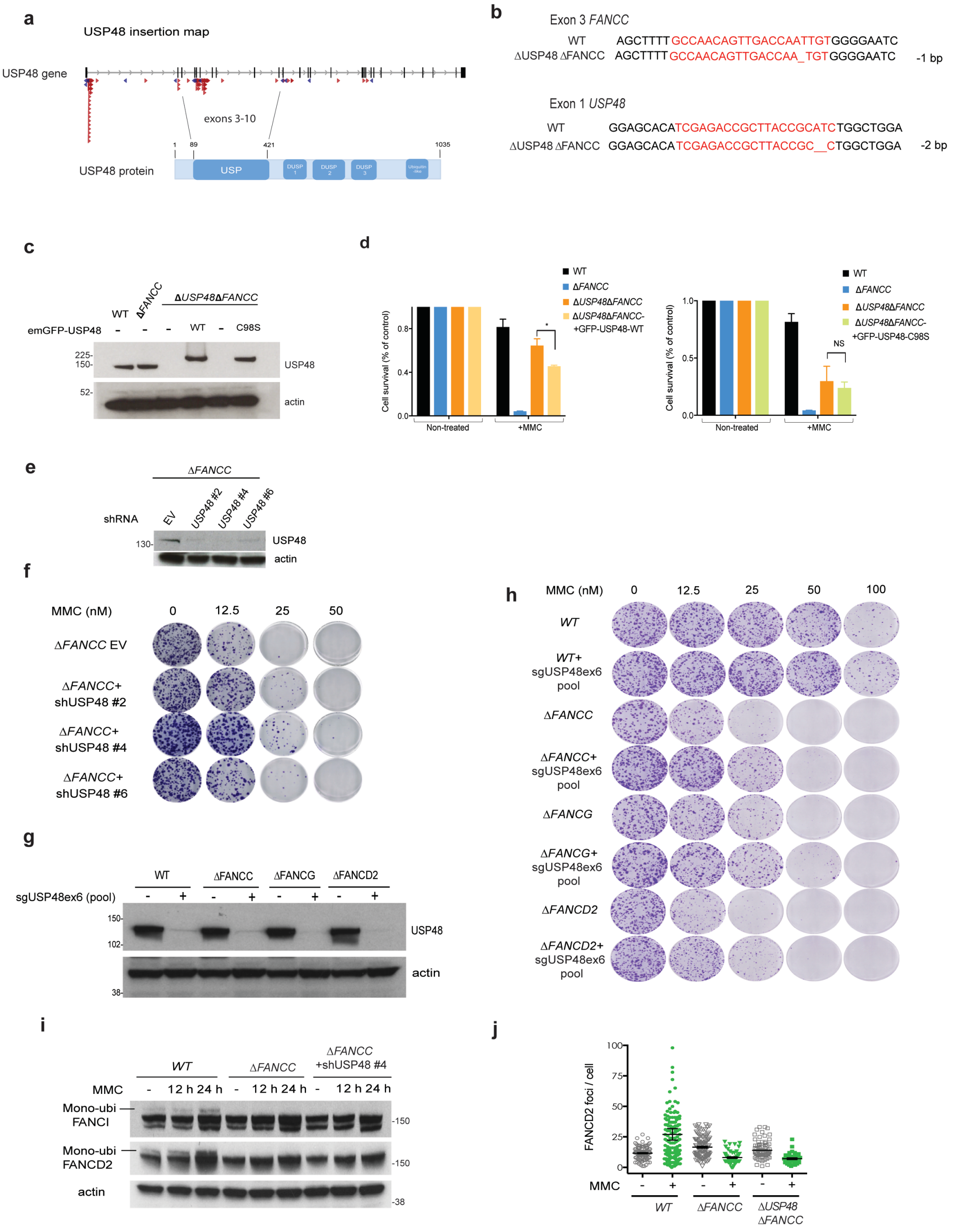

Supplementary Figure 2. Validation of suppression interaction between FA and USP48. (a) Gene-trap insertions within the *USP48* gene enriched in Δ*FANCC* cells treated with MMC. Red arrows indicate mutagenic insertions in the sense orientation while blue arrows indicate insertions in the antisense orientation (inactivating only in exonic regions). Schematic representation of the USP48 protein shows that the exonic region of exons 3 to 10 corresponds to the USP catalytic domain of the protein (source: Uniprot, Q86UV5 (UBP48_HUMAN). (b) CRISPR-Cas9-mediated mutation of FANCC and *USP48* in HAP1 cells. Red sequences in wild-type (WT) correspond to the gRNAs. *ΔUSP48* cells were purchased from Horizon Genomics. (c) Immunoblot for USP48 and actin. Higher band on the USP48 blot corresponds to the fused protein emGFP-USP48 WT/C98S. (d) Quantification of

viability of cells with reconstituted USP48 WT or USP48 catalytic inactive (C98S mutant), after treatment with MMC (25nM) for 6 days. Statistical significance was determined using a multiple t-test with $p<0.05$ as a threshold. *=$p<0.05$. Error bars indicate SD (standard deviation) of two replicates (e) Immunoblot for USP48 and actin. (f) Colony formation of *ΔFANCC* cells with or without *USP48* knockdown by shRNA 7 days after treatment with MMC at the indicated doses. (g) Immunoblot for USP48 and actin in FA deficient cells: *ΔFANCC, ΔFANCG* and *ΔFANCD2.* (h) Colony formation of *ΔFANCC, ΔFANCG* and *ΔFANCD2* cells with or without sgUSP48ex6 and CRISPR-Cas9 expression 7 days after treatment with MMC at the indicated doses. Sg=Single-guide RNA. (i) Immunoblot for FANCD2, FANCI and actin. Upper band corresponds to mono-ubiquitylated form of FANCD2 or FANCI. (j) Quantification of FANCD2 foci for the indicated cell lines was performed following MMC treatment (50nM) for 5 hours, similarly to the Rad51 foci quantification described in materials and methods.

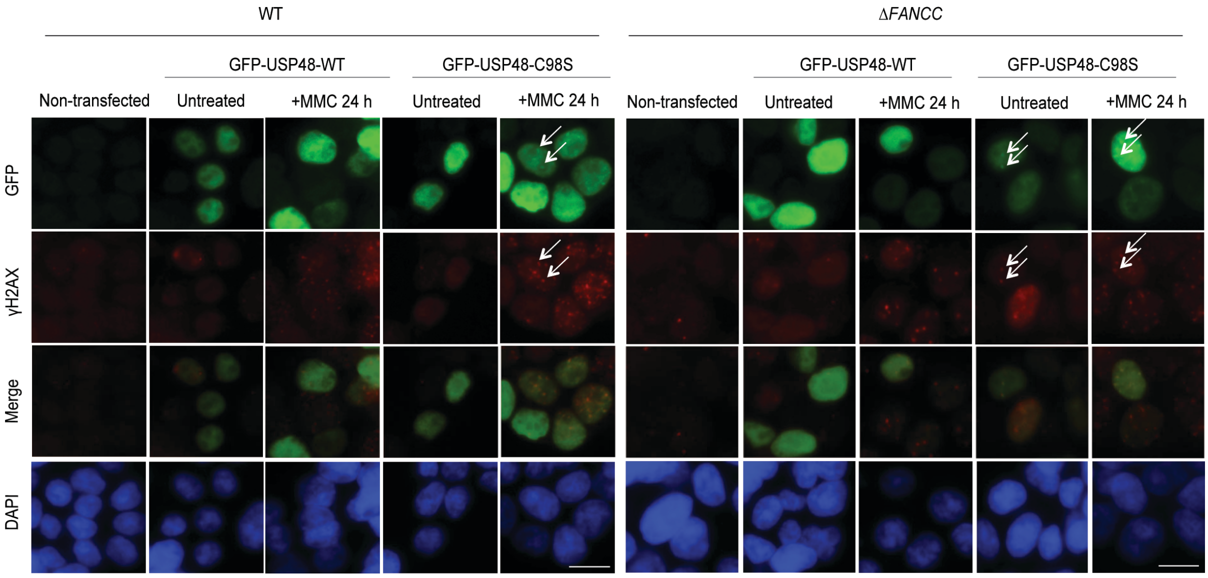

Supplementary Figure 3. GFP-USP48 is localized in the nucleus. Immunofluorescence images depicting the nuclear localization of GFP-USP48 WT and GFP-USP48-C98S after transfection in WT and *ΔFANCC* cells and the induction of γH2AX foci before and after MMC treatment (30nM) for 24 hours. Arrows indicate the colocalisation of GFP-USP48-C98S into foci with γH2AX. Scale bar=10μm.

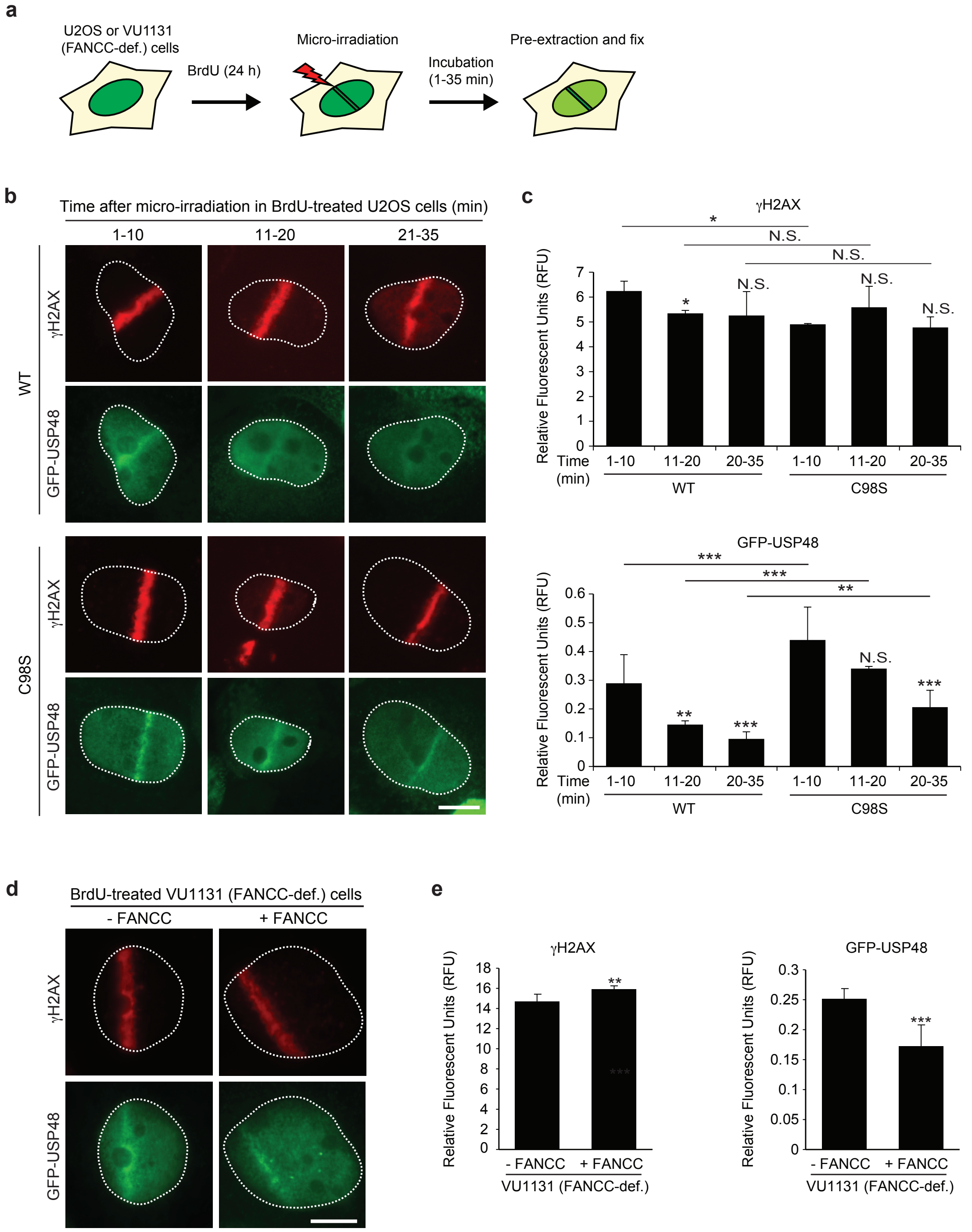

Supplementary Figure 4. USP48 is recruited to sites of DNA damage. (a) Workflow for the induction of DNA breaks and immunofluorescence in U2OS and VU1131 cells. (b-c) Images (b) and quantification (c) of γH2AX and GFP-tagged USP48 (WT and C98S mutant) localization to sites of laser micro-irradiation after BrdU sensitization in U2OS. N.S.=not significant; *=p<0.05; **=p<0.01; ***=p<0.001 (d-e) Images (d) and quantification (e) of γH2AX and GFP-tagged USP48 (WT and C98S mutant) localization to sites of laser micro-irradiation after BrdU sensitization in FANCC-deficient (VU1131) cells and FANCC-proficient (FANCC-complemented VU1131) cells. N.S.=not significant.; **=p<0.01; ***=p<0.001. Scale bar=10μm.

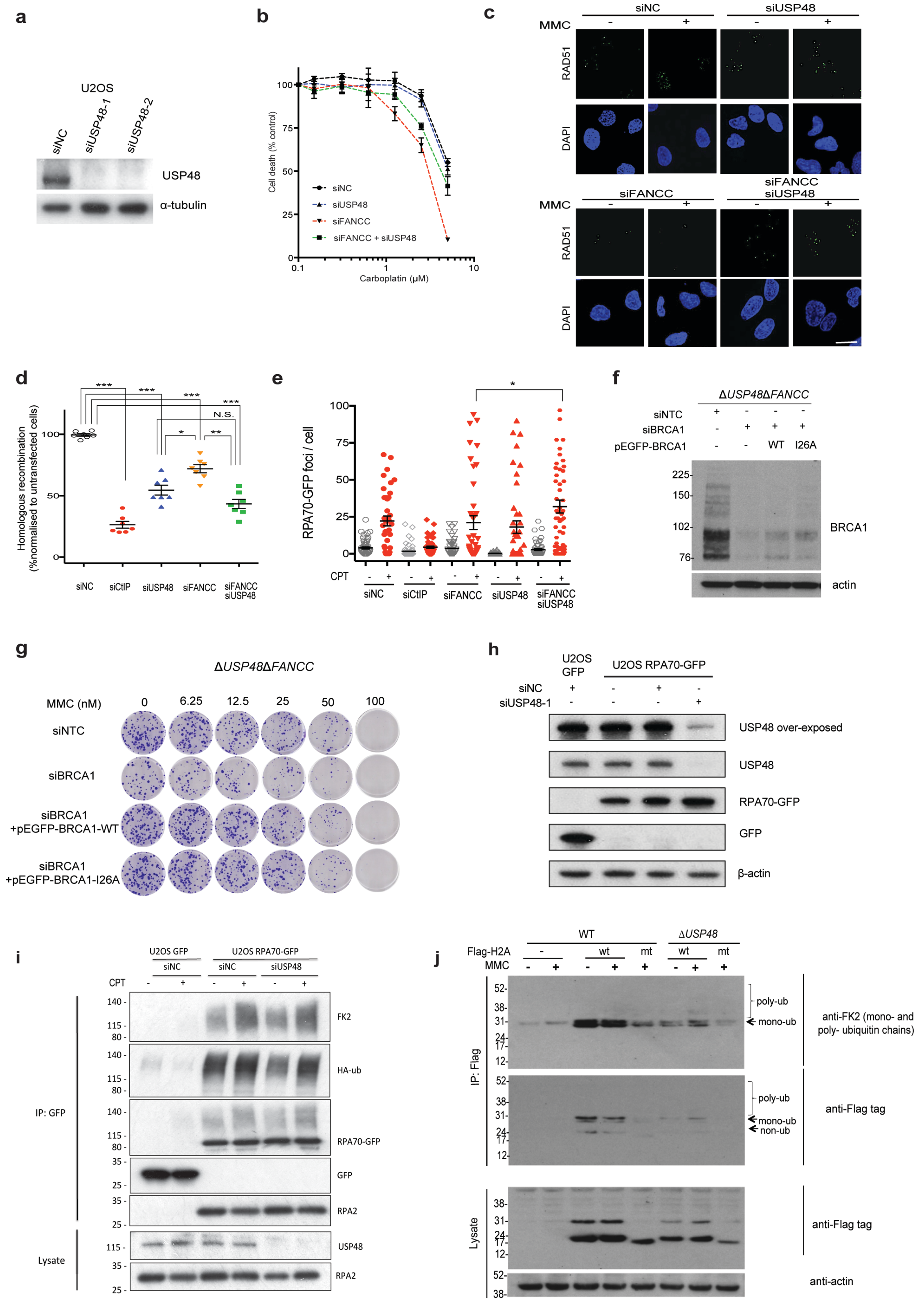

Supplementary Figure 5. Role of USP48 in the DNA damage response. Knockdown efficiency (a) and evaluation of sensitivity (b) of siNC (negative control), siUSP48, siFANCC and siUSP48siFANCC U2OS cells following Carboplatin exposure at the different doses. (c) Representative

immunofluorescence images after staining for RAD51 on WT, *siUSP48, siFANCC* and *siUSP48siFANCC* U2OS cells treated with MMC (100nM) for 18 hours. Scale bar=10µm (d) Quantification of HR repair after DSB induction with ISce-I enzyme, using the Traffic Light Reporter System developed in U2OS cells. N.S.=not significant; *=p<0.05; **=p<0.01; ***=p<0.001. (e) Quantification of RPA70-GFP foci for the indicated cell lines after treatment with Camptothecin (CPT; 1 µM) for 1h by the FACS-based RPA chromatinization assay as described in materials and methods. *=p<0.05. (f) Immunoblot for BRCA1 and actin. (g) Colony formation assay of *ΔUSP48ΔFANCC* cells with siRNA BRCA1 mediated knockdown and reconstitution of BRCA1 WT and BRCA1 ubiquitin ligase mutant (I26A) 7 days after treatment with MMC at the indicated doses. (h) Anti-GFP immunoprecipitation probed against USP48, RPA70-GFP, GFP and actin to determine knock-down efficiency. (i) anti-GFP immunoprecipitation blot probed against FK2, HA-ub, RPA70-GFP, GFP and RPA2; lysate blotted against USP48 and RPA2. (j) Anti-Flag H2A immunoprecipitation probed against FK2, Flag and actin for the indicated cell lines. Lower bands correspond to mono-ubiquitination modifications and higher bands correspond to poly-ubiquitin chains. Cells were transfected with wt=wild-type and mt=mutant (K5-9-118-119-125-127-129R) H2A and then treated with MMC (50nM) for 4 hours.

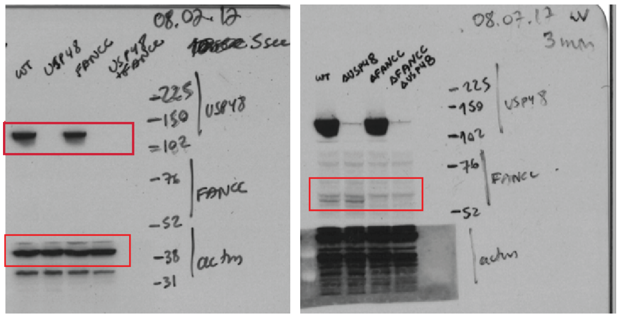

Figure 3a

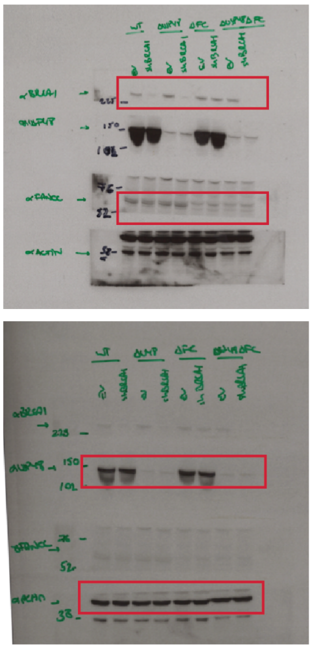

Figure 6a

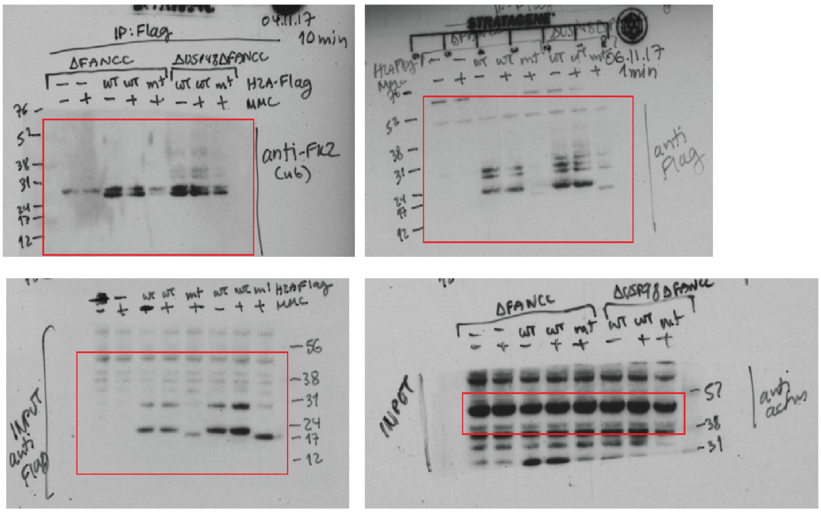

Figure 6c

Supplementary Figure 6. Uncropped Western Blot Films.
