# Supplementary material for: Map of synthetic rescue interactions for the Fanconi anemia DNA repair pathway identifies USP48
Source: Nat Commun. 2018 Jun 11;9:2280. doi: 10.1038/s41467-018-04649-z (PMC5996029; doi:10.1038/s41467-018-04649-z)
Supplement: Supplementary file 3 — Description of Additional Supplementary Files [file 41467_2018_4649_MOESM3_ESM.pdf]

Description of Additional Supplementary Files

File Name: Supplementary Data 1
Description: **List of enriched cellular processes specific to the FA-deficient cells.** Sheets 1-6 of the Excel file indicate the 5 independent clusters that have been identified through Network analysis and the enriched cellular processes that conform each cluster. Sheet 7 lists DNA repair genes that have been associated with the Hits retrieved from the gene-trap screens.

File Name: Supplementary Data 2
Description: **List of synthetic rescue genes.** List of protein-coding genes that have been identified to be rescue genes in the five different gene-trap screens (background mutations), with an fdr-corrected pvalue (q-value) < 0.05.
